# Far-ultraviolet light causes direct DNA damage in human lung cells and tissues

**DOI:** 10.1038/s41598-025-02869-0

**Published:** 2025-05-24

**Authors:** Asta Valanciute, Calum A. Ross, Alex Burden, Syam Mohan P. C. Mohanan, Mohammed Sabbah, Jessica M. W. Ghobrial, Stuart Dickson, Hui Xin Loh, Elvira Williams, Aysha Ali, Christian Brahms, Ahsan R. Akram, Lynn Paterson, Natalie K. Jones, Bethany Mills, John C. Travers, Robert R. Thomson, Kevin Dhaliwal

**Affiliations:** 1 Centre for Inflammation Research, Institute for Regeneration and Repair, University of Edinburgh, Edinburgh, UK; 2 Institute of Photonics and Quantum Sciences, Heriot-Watt University, Edinburgh, UK; 3 Institute of Biological Chemistry, Biophysics and Bioengineering, Heriot-Watt University, Edinburgh, UK

**Keywords:** Far-UVC, 222 nm, 206 nm, DNA damage, (6-4) PP, H2AX(Ser139), Cell death, Human lung cells, Biotechnology, Cell biology, Medical research, Physics

## Abstract

Far-ultraviolet C (Far-UVC) radiation, with a wavelength range from 200 to 235 nm, is germicidal and holds potential for clinical applications. However, its use against deep-seated and internal infections, such as those affecting the lungs, remains less well established. The safety profile of Far-UVC irradiation requires further investigation across different human tissues. In this study, we utilised a krypton-chloride excimer lamp and a pulsed laser system to examine the effects of Far-UVC irradiation on human lung cells in vitro and primary human tracheal tissue. Primary human tracheal tissue and cells exposed to continuous wave (222 nm) and pulsed 206 nm and 222 nm light at doses of 5, 25, and 50 mJ/cm^2^ exhibited DNA damage, including phosphorylation of γH2AX (Ser139). The continuous wave and pulsed 222 nm irradiation caused the formation of pyrimidine-pyrimidone (6-4) photoproducts. Irradiated human lung cells demonstrated reduced viability in vitro, and increased lactate dehydrogenase release into the culture medium 48 h post-irradiation. Our findings reveal that even low doses of Far-UVC (206 nm, 222 nm) light can penetrate monolayers of human lung epithelial cells, causing direct DNA damage in the form of (6 -4) photoproducts and DNA double-strand breaks, ultimately leading to cell death.

**Supplementary Information:**

The online version contains supplementary material available at 10.1038/s41598-025-02869-0.

## Introduction

Ultraviolet-C (UVC) light, with wavelengths ranging from approximately 200 nm to 280 nm, is widely recognised for its potent germicidal properties. Recently, the potential for Far-UVC light (200–235 nm) to reduce the transmission of airborne pathogens such as SARS-CoV-2 has been extensively studied^1–4^ At lower doses (6, 17 and 24 mJ/cm^2^), 222 nm light has been shown to inactivate various bacteria, including *Staphylococcus aureus*, *Pseudomonas aeruginosa* and *Escherichia coli*^5–7^ The germicidal mechanism of 222 nm light is attributed to its ability to degrade aromatic amino acids, oligopeptides, proteases, and proteins, and to damage DNA^8^.

UVC radiation induces direct Deoxyribonucleic acid (DNA) damage through the formation of a variety of premutagenic and cytotoxic DNA lesions, including pyrimidine dimers. These pyrimidine dimers can be further categorized into cyclobutene pyrimidine dimers (CPDs) and pyrimidine-pyrimidone (6 − 4) photoproducts ((6 − 4) PPs). Additionally, UVC radiation absorbed by nucleic acids and proteins can generate oxidative DNA damage, referred to as indirect DNA damage^9,10^.

Despite its highly mutagenic and efficient germicidal activity against viruses and bacteria, 222 nm light has been reported to be non-harmful to mammalian skin and eye cells under certain conditions^11–16^ However, recent studies have revealed that exposure to 222 nm irradiation can result in significant DNA double-strand breaks (DSBs) in human retinal cell lines (ARPE-19) and HEK-A keratinocytes *in vitro*^17^ Furthermore, irradiation of monolayered colon epithelial cells with 30 mJ/cm^2^ of 222 nm wavelength was found to compromise cell membrane integrity and reduce cell viability^18^.

The efficacy and safety of Far-UVC light in addressing pulmonary infections, such as ventilator-associated pneumonia caused by multidrug-resistant bacteria or early-stage chronic lung conditions, remain less well-studied. We hypothesised that catheter-delivered Far-UVC irradiation could serve as a potential alternative for treating antibiotic-resistant bacterial infections in the airways. However, no comprehensive studies have been conducted to evaluate the molecular alterations induced by Far-UVC irradiation in human lung epithelial cells or lung tissue. Furthermore, the biological effects of exposure to low doses of continuous wave (CW) or pulsed Far-UVC irradiation on human lung epithelial cells remain unexplored.

The primary objective of this study was to investigate the biological effects of 206 nm and 222 nm irradiation on DNA damage in human lung cells, with the aim of assessing its safety and potential as a tool for a wide range of clinical applications.

## Results

### 222 nm-induced DNA DSBs in monolayered human cells

Previous studies have shown that UV-B (280–315 nm)^10,19^ and Far-UVC wavelengths: 206 nm^20^ and 222 nm^17^ can penetrate cells, traverse the cytoplasm, reach the nucleus, and induce DNA damage, leading to an excess of DNA double-strand breaks.

**Fig. 1:**
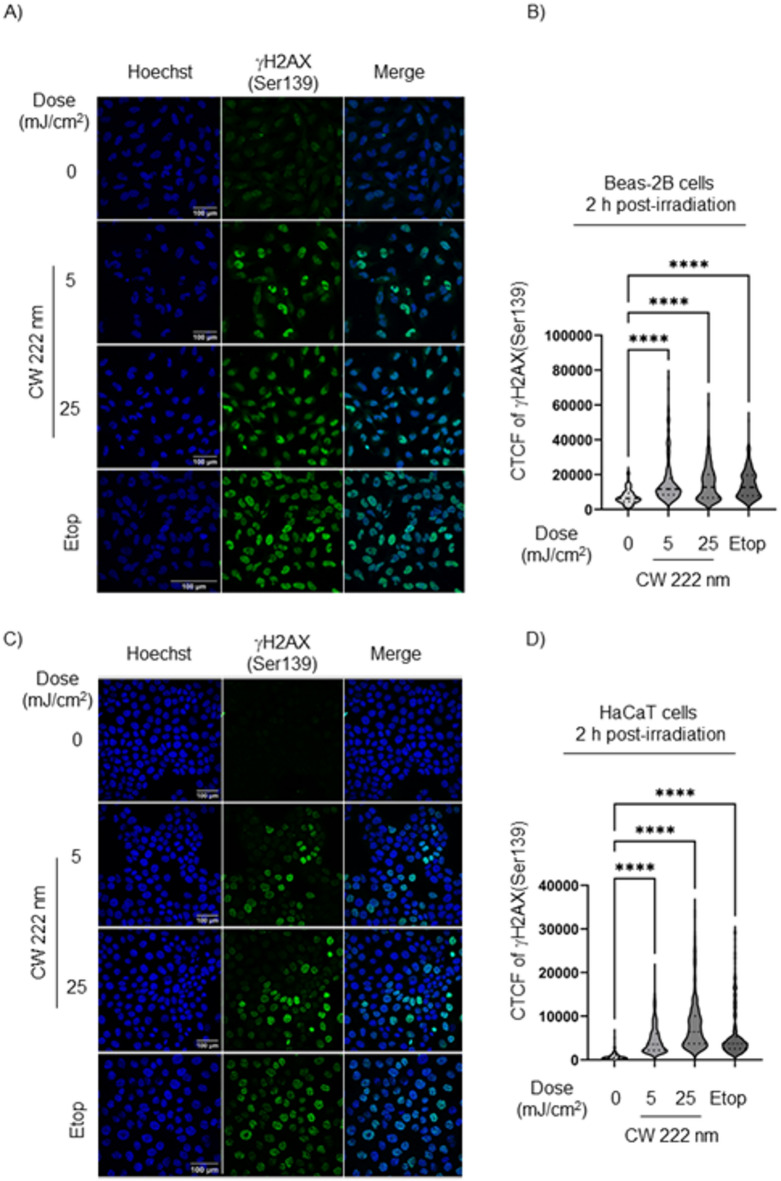
CW 222 nm-induced DNA DSBs damage in monolayered Beas-2B and HaCaT cells *in vitro*. (**A**) Representative immunofluorescence images of  γH2AX(Ser139) staining (green) in Beas-2B cells 2 hours post-irradiation with CW 222 nm. Hoechst nuclei (blue). An untreated sample (0 mJ/cm^2^) was used as an internal control. Etop was used as a positive control of   γH2AX(Ser139) staining. Scale bar, 100 μm. (**B**) Quantification of the   γH2AX(Ser139) staining as the corrected total cell fluorescence (CTCF) was performed on Beas-2B cells 2 hours post-irradiation. Data presented as means ± SD from *n* = 3 experiments. In total 306 cells were analyzed per condition. One-way ANOVA with repeated measures (****p < 0.0001). (**C**) Representative immunofluorescence images of   γH2AX(Ser139) staining (green) in HaCaT cells at 2 hours following CW 222 nm irradiation. Hoechst nuclei (blue). Scale bar, 100 μm. (**D**) Quantification of the    γH2AX(Ser139) activation was performed on HaCaT cells 2 hours post-irradiation with CW 222 nm. Data presented as means ± SD from *n* = 3 experiments. In total 361 cells were analyzed per condition. One-way ANOVA with repeated measures (****p < 0.0001).

We investigated DNA DSBs damage in human cells, irradiated with CW 222 nm light at doses of 5 and 25 mJ/cm^2^, using immunofluorescence staining for γH2AX(Ser139) 2 h after irradiation. Irradiated Beas-2B cells exhibited a dose-dependent increase in DNA DSBs, as evidenced by γH2AX(Ser139) staining. Hoechst staining revealed no nuclear aggregation or fragmentation in the irradiated Beas-2B cells (Fig. 1A). Furthermore, CW 222 nm exposure resulted in significantly higher γH2AX(Ser139) fluorescent intensity compared to non-irradiated controls (Fig. 1B). Similarly, CW 222 nm irradiation increased γH2AX(Ser139) phosphorylation in HaCaT keratinocytes (Fig. 1C,D), a skin cell model known for its resistance to Far-UVC light exposure.

**Fig. 2:**
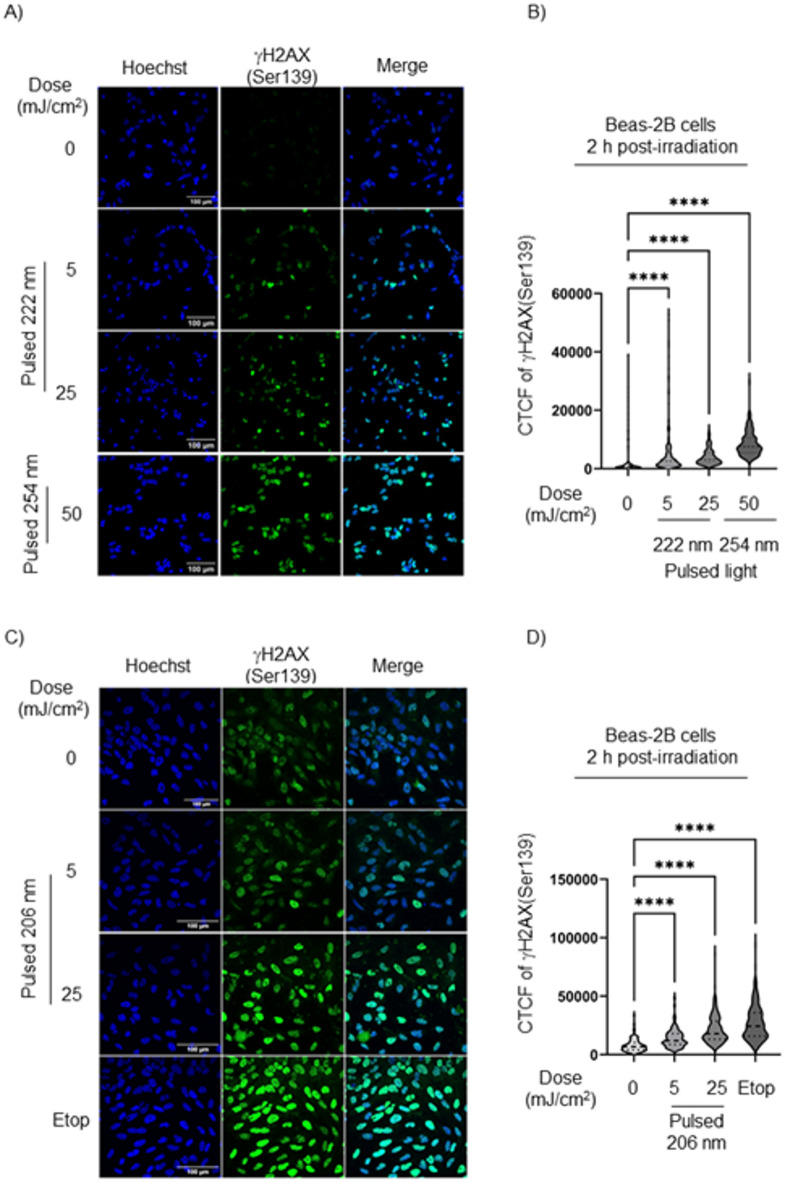
Pulsed 222 nm and 206 nm irradiation induced DNA DSBs damage in monolayered Beas-2B *in vitro*. (A) Representative immunofluorescence images of   γH2AX(Ser139) staining (green) in Beas-2B cells 2 hours post-irradiation with pulsed 222 nm. Hoechst nuclei (blue). Pulsed 254 nm light (50 mJ/cm^2^) was used as a positive control of  γH2AX(Ser139) staining. Scale bar, 100 μm. (B) Quantification of the  γH2AX(Ser139) staining as the corrected total cell fluorescence (CTCF) was performed on Beas-2B cells 2 hours post-irradiation. Data presented as means +/-SD from *n* = 3 experiments. In total 237 cells were analyzed per condition. One-way ANOVA with repeated measures (****p < 0.0001). (C) Representative immunofluorescence images of  γH2AX(Ser139) staining (green) in Beas-2B cells at 2 hours following pulsed 206 nm irradiation. Hoechst nuclei (blue). Scale bar, 100 μm. (D) Quantification of the  γH2AX(Ser139) activation was performed on Beas-2B cells 2 hours post-irradiation with pulsed 206 nm. Data presented as means +/-SD from *n* = 3 experiments. In total 232 cells were analyzed per condition. One-way ANOVA with repeated measures (****p < 0.0001).

We further evaluated the DNA damage response in pulsed 222 nm- and 206 nm-irradiated Beas-2B cells *in vitro*. Following 5 and 25 mJ/cm^2^ of pulsed 222 nm and 206 nm irradiation, monolayer-cultured Beas-2B cells showed a dose-dependent increase in γH2AX(Ser139) phosphorylation compared to non-irradiated cells (Fig. 2A–D, respectively).

### 222 nm-induced DNA double-strand breaks and (6-4) PPs in human trachea

**Fig. 3:**
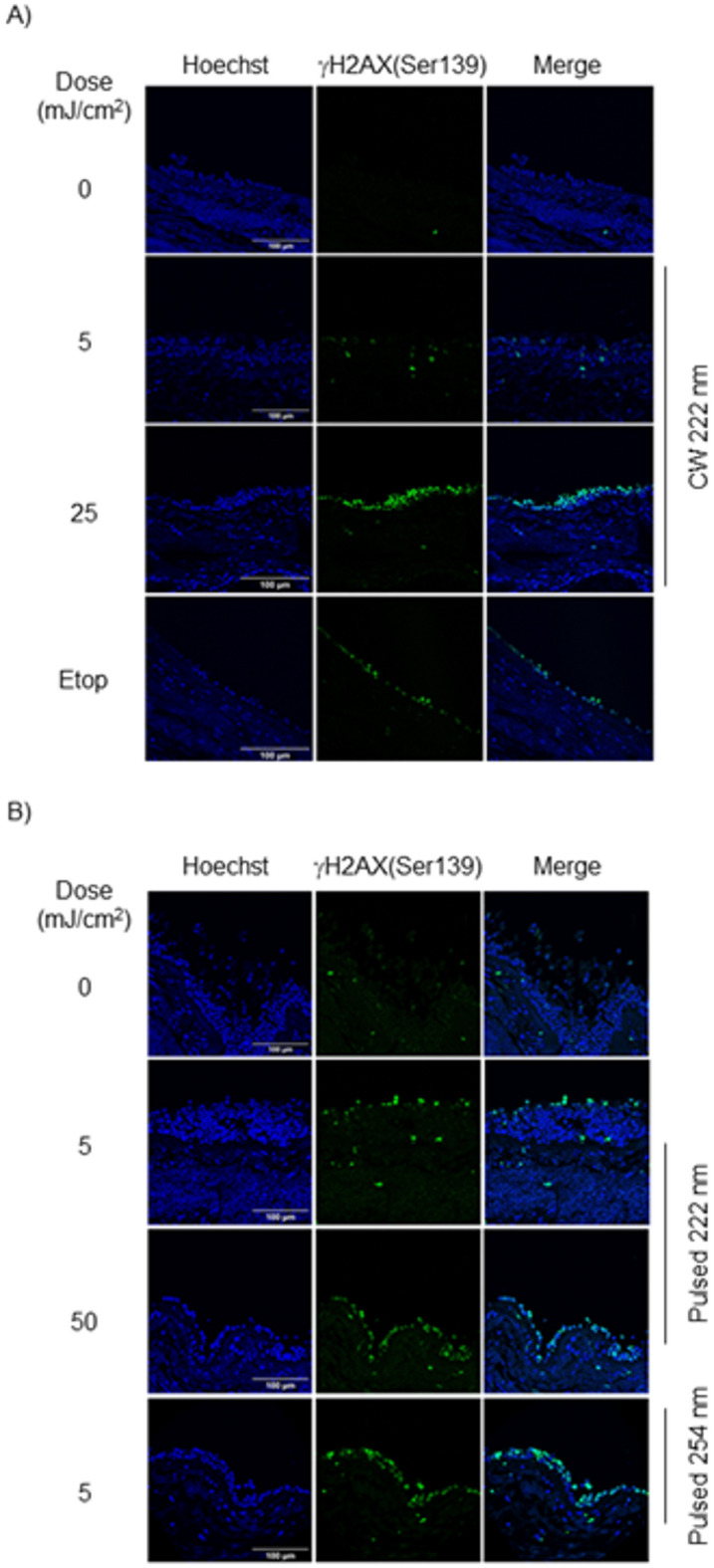
CW and pulsed 222 nm irradiation induced DNA DSBs in human trachea. (**A**) Representative immunofluorescence images of  γH2AX(Ser139) staining (green) in human trachea after CW 222 nm irradiation. Hoechst nuclei (blue). Scale bar, 100 μm. (**B**) Representative immunofluorescence images of  γH2AX(Ser139) staining (green) after pulsed 222 nm irradiation in human trachea. Hoechst nuclei (blue). Pulsed 254 nm (5 mJ/cm^2^) irradiation was used as a positive control. Scale bar, 100 μm.

Building on our findings of 222 nm-induced DNA DSBs in monolayer-cultured cells 2 h after irradiation, we hypothesised that DNA in the ciliated pseudostratified columnar epithelium of the human trachea could also be damaged by 222 nm irradiation. Using fluorescent confocal microscopy and γH2AX(Ser139) staining, we observed that CW 222 nm, pulsed 222 nm and pulsed 206 nm irradiation, at doses of 5, 25, and 50 mJ/cm^2^, resulted in a significantly higher number of γH2AX(Ser139)-positive cells compared to non-irradiated tissue 10 min after the initial irradiation (Fig. 3A,B, S1A,B, respectively).

**Fig. 4:**
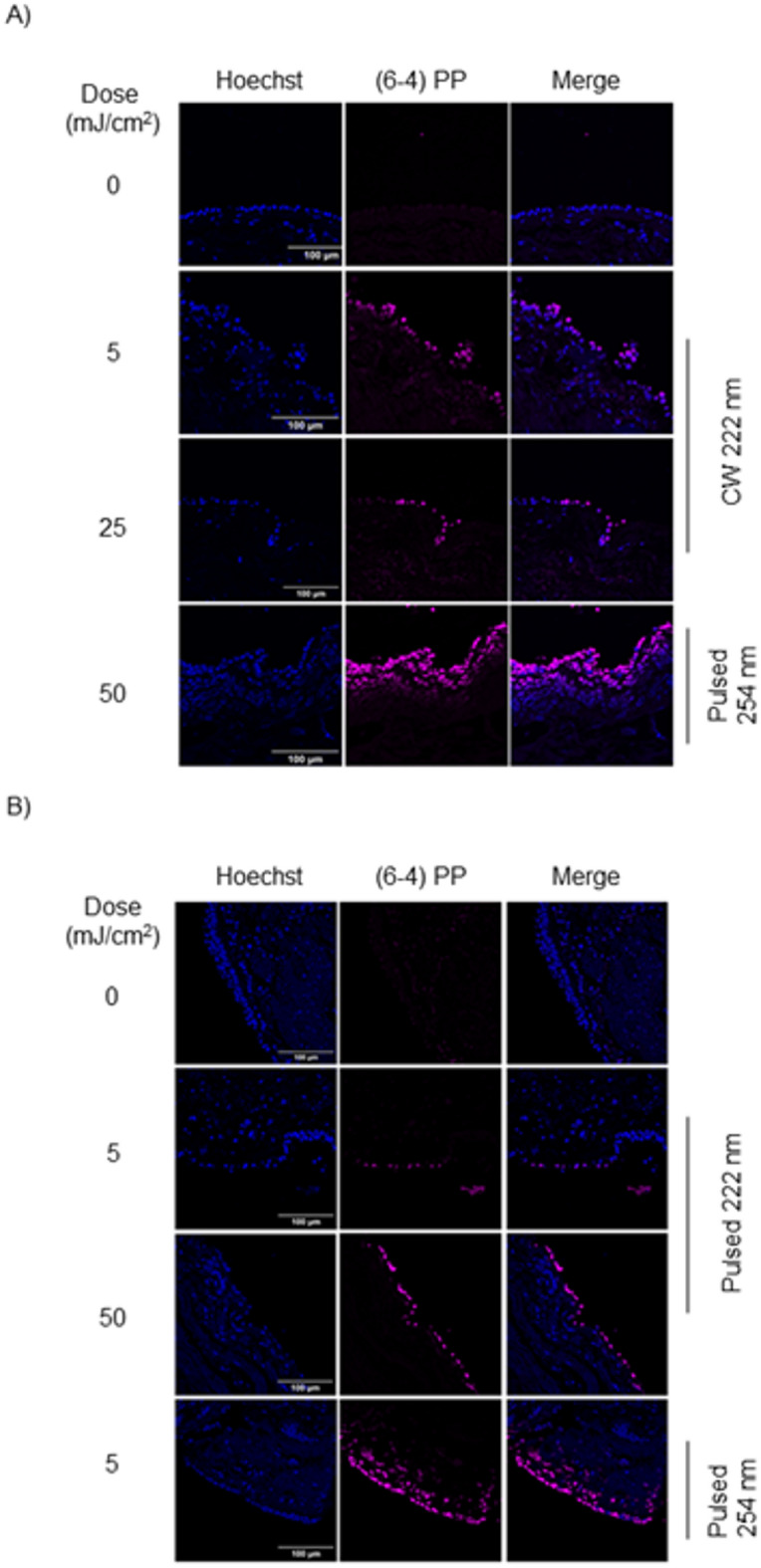
(6-4) PP-expressing cells were detected in human trachea after 222 nm irradiation. (**A**) Representative immunofluorescence images of (6-4) PP staining (magenta) in human trachea 10 min after CW 222 nm irradiation. Hoechst nuclei (blue). Pulsed 254 nm-irradiated samples were used as a positive control. Scale bar, 100 μm. (**B**) Representative immunofluorescence images of (6-4) PP staining (magenta) in human trachea 10 min after exposure to pulsed 222 nm light. Hoechst nuclei (blue). Scale bar, 100 μm.

In addition to causing DNA DSBs, UVC radiation also generates thousands of DNA lesions in living cells,^21,22^ for example, (6-4) PPs. We investigated the formation of (6−4) PPs in human tracheal tissue following CW and pulsed 222 nm irradiation at energy doses of 5, 25, and 50 mJ/cm^2^. Both CW and pulsed 222 nm irradiation induced (6−4) PPs formation in the upper epithelial cells of the trachea in a dose-dependent manner, observed 10 min after irradiation (Fig. 4A,B, and Fig. S2).

### Impact of 222 nm light on cell viability and apoptosis in human cells *in vitro*

**Fig. 5:**
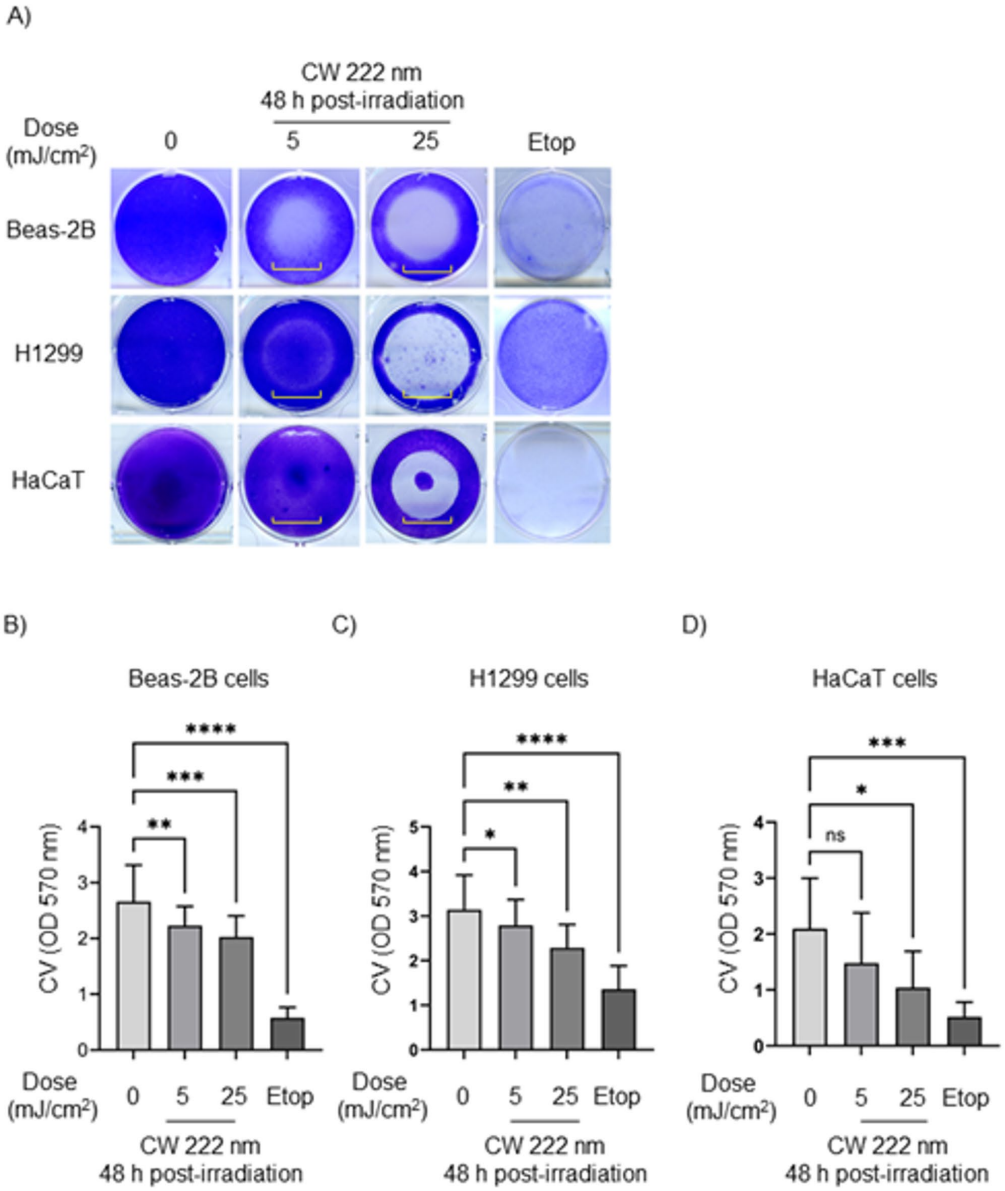
CW 222 nm irradiation resulted in decreased cell viability in human cells *in vitro* 48 h post-irradiation. (**A**) Representative images of CV staining on Beas-2B, H1299 and HaCaT cells 48 h after CW 222 nm irradiation. A square yellow bracket indicates an irradiated area. (**B**) The OD of CV-stained Beas-2B cells was measured at 570 nm. Data are shown as mean ± SD from *n* = 3 experiments. (**C**) The OD of CV-stained H1299 was measured at 570 nm. Data are presented as mean ± SD from *n* = 3 experiments (**D**) The OD of CV-stained HaCaT cells was measured at 570 nm Data are presented as mean ± SD from *n* = 3 experiments. Where applicable, one-way ANOVA with repeated measures was performed (ns, not significant, *p < 0.05, **p < 0.01, ***p < 0.001, ****p < 0.0001.

After identifying significant DNA damage in human lung cells exposed to 222 nm light, we explored cell survival 48 h post-irradiation. After CV staining, we localized the irradiation spot in the human adherent cells in vitro, also we observed that two human lung cell lines (Beas-2B and H1299) and HaCaT keratinocytes subjected to CW 222 nm irradiation failed to proliferate within 48 h of exposure. At this time point, a distinct irradiated area with reduced CV staining intensity (highlighted by a yellow square bracket) was evident in all three CW 222 nm-irradiated cell lines (Fig. 5A). Optical density measurements of CV at 570 nm confirmed that CW 222 nm irradiation induced cytotoxicity in a dose-dependent manner across all three cell lines (Fig. 5B–D, respectively).

**Fig. 6:**
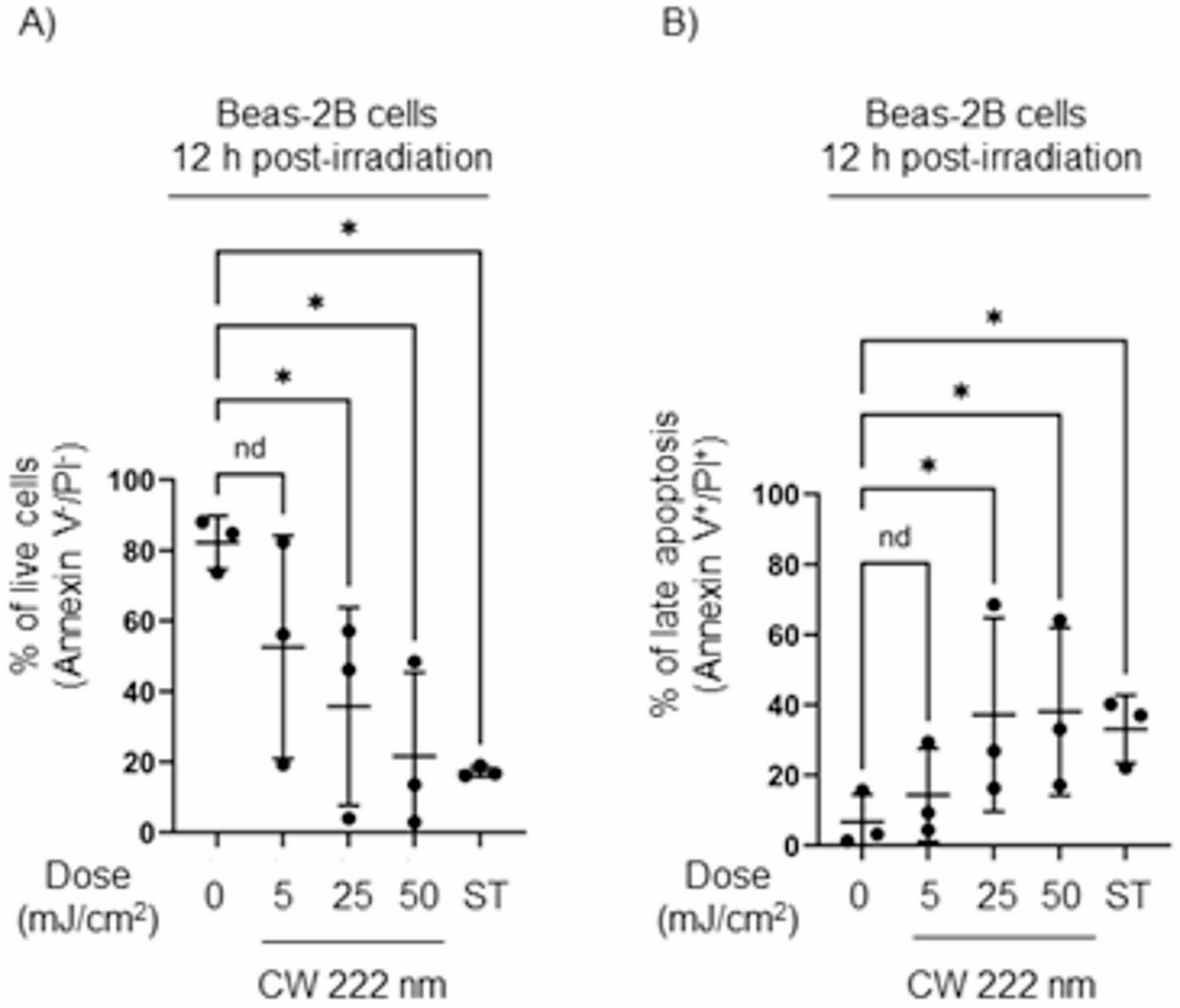
CW 222 nm irradiation reduced cell viability and induced apoptosis 12 h post-irradiation in Beas-2B cells. (**A**) Decreased Beas-2B cell viability was demonstrated by FITC Annexin V and PI assay via flow cytometry 12 h post-irradiation. Data presented as means ± SEM from *n* = 3 experiments with representative flow cytometry dot plots per treated group. (**B**) Late apoptosis demonstrated in Beas-2B cells 12 h post-irradiation by Annexin V assay. Data presented as means ± SEM from *n* = 3 experiments with representative flow cytometry dot plots per treated group. Were applicable, data (**A**) and (**B**) was analysed using the Friedman test (nd, not a discovery, *p < 0.05).

After identifying a decreased proliferation and survival of CW 222 nm-irradiated cells 48 h post-irradiation, we next interrogated whether CW 222 nm-irradiated cells show apoptosis at earlier timepoint (12 h post-irradiation). Using FITC Annexin V and PI staining with flow cytometry, we found that Beas-2B cells irradiated with CW 222 nm light showed reduced viability compared to non-irradiated cells 12 h post-irradiation (Fig. 6A). Specifically, CW 222 nm irradiation at doses of 25 and 50 mJ/cm^2^ resulted in a significantly decrease percentage of live cells (36% and 30%, respectively) compared to non-irradiated cells (82%) (Fig. 6A). This reduction correlated with increased late apoptosis, as indicated by positive Annexin V and PI staining (Fig. 6B).

**Fig. 7:**
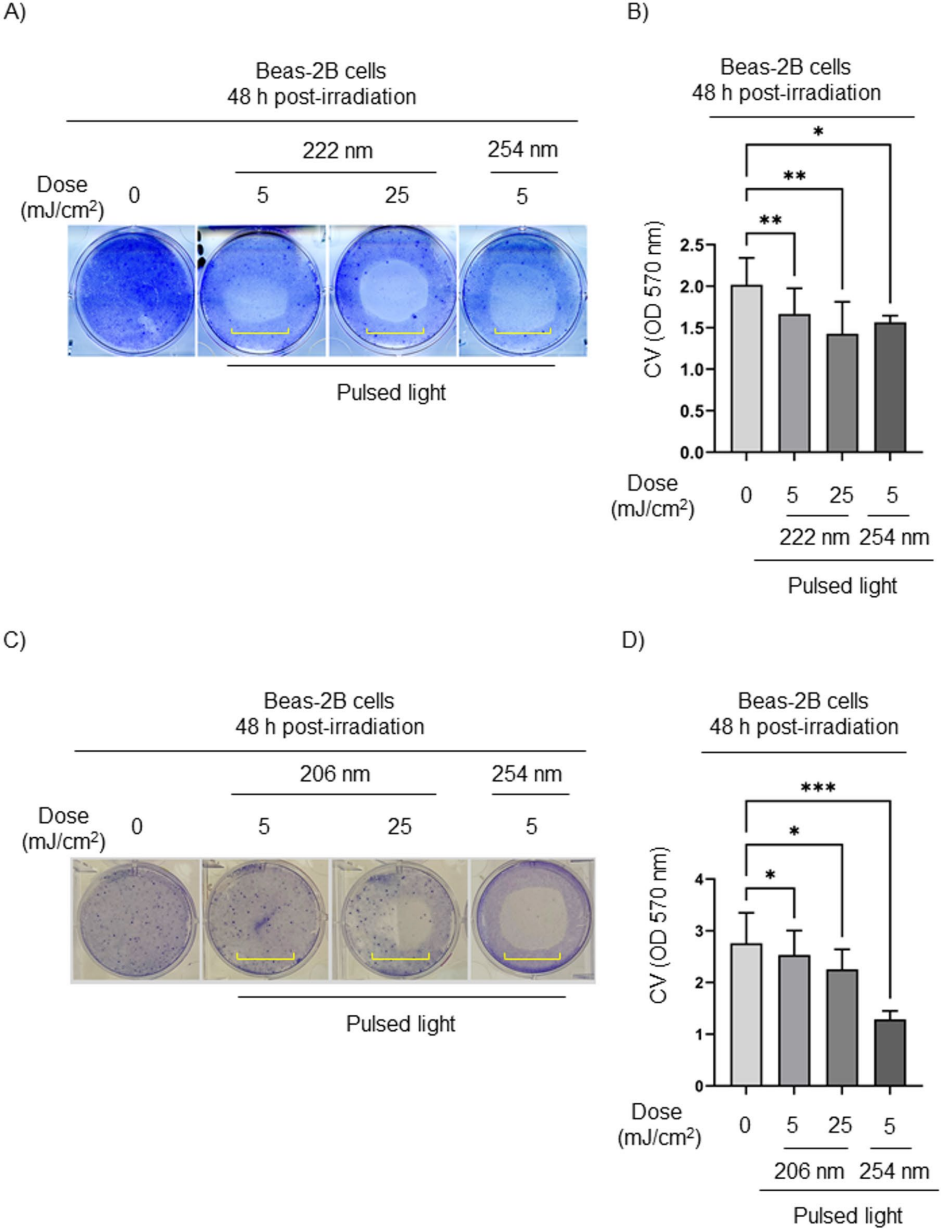
Decrease of CV staining in pulsed 222 nm- and 206 nm-irradiated Beas-2B cells. (**A**) Representative images of CV staining on Beas-2B cells at 48 h after pulsed 222 nm irradiation. A square yellow bracket indicates an irradiated area. (**B**) The OD of CV-stained Beas-2B cells 48 h post-irradiation with pulsed 222 nm light. Data are shown as mean ± SD from *n* = 3 experiments (**C**) Representative images of CV-stained Beas-2B cells 48 h after pulsed 206 nm irradiation. A square yellow bracket indicates an irradiated area. (**D**) The OD of CV-stained-Beas-2B cells 48 h post-irradiation with pulsed 206 nm light. Data are shown as mean ± SD from *n* = 3 experiments. Where applicable, one-way ANOVA with repeated measures was performed (*p < 0.05, **p < 0.01, ***p < 0.001).

After determining the cell-killing effect of low-dose CW 222 nm irradiation *in vitro*, we next evaluated whether both pulsed 222 nm and 206 nm lights could reduce cell viability 48 h post-irradiation. Pulsed 222 nm irradiation decreased the viability of Beas-2B cells, as indicated by reduced CV staining (highlighted by a yellow square bracket) at 48 h post-exposure. Beas-2B cells exposed to pulsed 222 nm irradiation (5, 25 mJ/cm^2^) did not survive and detached from the plate, whereas non-irradiated cells remained viable (Fig. 7A). Optical density measurements of CV staining confirmed the cytotoxic effects of pulsed 222 nm irradiation in a dose-dependent manner, inhibiting the expansion of irradiated cells 48 h post-exposure (Fig. 7B). In concordance with CW 222 nm and pulsed 222 nm irradiation, pulsed 206 nm irradiation also decreased viability of Beas-2B cells 48 h post-irradiation (Fig. 7C,D).

**Fig. 8:**
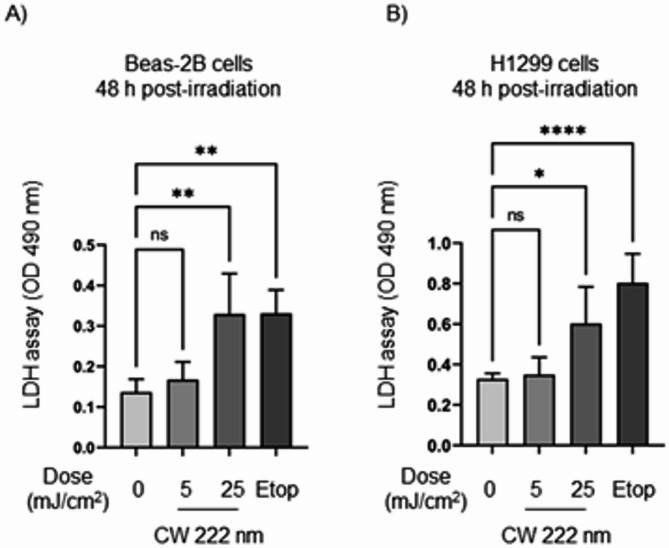
CW 222 nm irradiation induced an increase of lactate dehydrogenase into cell culture media 48 h post-irradiation. The extracellular LDH levels in the culture media from CW 222 nm-irradiated (**A**) Beas-2B and (**B**) H1299 cells measured at 490 nm. Data presented as means ± SD from *n* = 3 experiments. One-way ANOVA with repeated measures (ns, not significant, *p < 0.05, **p < 0.01, ****p < 0.0001).

It has also been shown that UVC irradiation can damage the cell membrane leading the cell content to leak into the cell medium^23^ A lactate dehydrogenase cytotoxicity assay was performed to investigate possible cell membrane damage after CW 222 nm irradiation. We found that CW 222 nm irradiation damaged the cell membrane in Beas-2B and H1299 cells 48 h post-irradiation at a dose of 25 mJ/cm^2^ compared to non-irradiated cells as indicated by an increase in LDH release into the culture medium (Fig. 8A,B, respectively).

## Discussion

Far-UVC light with a wavelength of 222 nm has been reported as an effective antimicrobial and antiviral agent^1,2,5^ In recent years, multiple studies have investigated its impact on skin models, concluding that 222 nm irradiation offers high germicidal efficacy with minimal safety concerns compared to longer UVC wavelengths such as 254 nm^11,24,25^. If 222 nm light can inactivate antibiotic-resistant bacteria while being well-tolerated by human cells without inducing DNA damage or photocarcinogenesis, it could be a promising option for clinical applications to prevent colonisation and treat antibiotic-resistant pathogens in the airways. However, a key issue remains determining the safety of Far-UVC irradiation on human lung cells and tissues.

Recent studies have shown that CW 222 nm irradiation at a dose of 87.6 mJ/cm^2^ increased the percentage of γH2AX-positive cells and reduced proliferation in human retinal pigment epithelial cells compared to non-irradiated controls^17^ In our study, we found that CW 222 nm, pulsed 222 nm and pulsed 206 nm irradiation (5, 25, 50 mJ/cm^2^) induced γH2AX phosphorylation at Ser139, a marker of DNA DSBs, in a dose-dependent manner in human lung cells and in human tracheal tissue.

Previous studies have demonstrated that 254 nm UV irradiation causes DNA lesions^12,15,19^ Unlike 254 nm, 222 nm irradiation has been assumed safe for skin and eye models without inducing CPDs or (6-4) PPs^12,13,26^ However, 311 nm irradiation (30 mJ/cm^2^) has been shown to generate (6-4) PPs in human skin fibroblasts, with (6 -4) PPs—rather than CPDs—playing a critical role in impeding DNA replication and leading to cell death^27^ In our study, we detected a strong upregulation of (6 -4) PPs in the human tracheal epithelium after CW and pulsed 222 nm exposure at low doses (5, 25, 50 mJ/cm^2^).

Prolonged, unrepaired DNA lesions and DNA DSBs following UV exposure can impair cell growth and lead to cell death. Both Ong et al.^17^ and Nishikawa et al.^18^ reported that 222 nm-irradiated cells exhibited increased apoptosis and reduced viability. In our study, we observed a significant decrease in cell viability 12- and 48-h post-irradiation, with no recovery, indicating continuous cell death beyond initial exposure.

We demonstrated both the short (10 min–2 h) and long-term (12 and 48 h) effects of Far-UVC irradiation on human lung cells and tracheal tissue. A single low-dose exposure (5, 25, 50 mJ/cm^2^) of CW 222 nm, pulsed 222 nm and pulsed 206 nm irradiation caused direct DNA damage in the nucleus, leading to cellular toxicity and decreased viability *in vitro*. These results provide clear evidence of toxicity, limiting any potential use of Far-UVC, including 206 nm and 222 nm wavelengths, for pulmonary applications.

## Methods

All chemicals, cell lines, media, antibodies, fluorescence probes, commercial assays, and tools used in this study are listed in Table S1 (Supplementary Information).

### Experimental sources of Far-UVC irradiation

In this study, we used two Far-UVC light sources to assess the effects of irradiation on human cells in vitro and tracheal tissue. The first source was a krypton-chloride (KrCl) excimer lamp (Ushio Care222 module; Table S1), a CW system equipped with filters to maintain a constant 222 nm output. This lamp was positioned 20 cm from the sample to ensure uniform UV intensity across an entire six-well plate, a setup used consistently in all experiments.

The second light source has been described by Brahms et al.^28^ It is an ultrafast pulsed laser system based on frequency conversion in gas-filled hollow capillary fibres, capable of tuning from 206 nm to 254 nm wavelengths. In this study, pulsed 254 nm irradiation served as a positive control for DNA damage induction in mammalian cells^19^ The pulsed source operated at a 10 kHz repetition rate, with its output beam expanded and collimated up to a diameter of ~ 20 mm before being directed onto individual samples in well plates.

To compare the impact of CW and pulsed light, the intensity of both sources at the sample was measured and adjusted to be approximately 0.28 mW/cm^2^. Total irradiation doses of 5, 25, and 50 mJ/cm^2^ were achieved by varying exposure times. Ozone generation from both CW and pulsed Far-UVC sources was considered minimal and insignificant due to the short exposure duration.

### Cell culture

Human lung cell lines, including Beas-2B (immortalised human bronchial epithelial cells), H1299 (human non-small cell lung carcinoma cells), and HaCaT (human keratinocytes), were obtained from ATCC (Manassas, VA, USA). Cells were cultured in complete medium consisting of DMEM supplemented with 10% fetal bovine serum (FBS), 100 IU/mL penicillin G sodium salt, 100 µg/mL streptomycin sulfate, and 2 mM L-glutamine. They were routinely passaged in T25 cell culture flasks upon reaching 80–90% confluence. Mycoplasma contamination was regularly tested, and all assays were conducted using mycoplasma-free cell cultures.

### Human trachea

Human lungs that were unsuitable for transplantation were obtained from the National Health Service Blood and Transplant (NHSBT). The studies involving this human tissue were approved by the London—Central Research Ethics Committee (REC No: 16/LO/1883) and supported by NHS Lothian SAHSC Bioresource (REC No: 20/ES/0061).

Trachea was excised from the donated lungs and washed with 50 mL of PBS solution and then cut into 1 cm^2^ pieces. Five hundred microliters of DMEM complete medium (without phenol red) were applied to the tissue. The trachea, with its airway ciliated cells exposed, was then directly irradiated with Far-UVC light.

### Immunofluorescence for human cells

Cells were seeded on coverslips and cultured for 24 h before exposure to 222 nm light, followed by an additional 2-h incubation. Cells were then fixed in 4% paraformaldehyde for 15 min and incubated in a permeabilization/blocking buffer (1% BSA, 10% FBS, 0.1% Triton X-100, 0.1% Saponin) for 1 h at room temperature (RT).

Primary antibody for γH2AX(Ser139) was diluted (1:200) and incubated overnight at 4 °C, followed by a 1-hour incubation with secondary antibody (Alexa Fluor^TM^488 anti-Rabbit IgG, 1:1000) and Hoechst 33342 (1:1000) at RT in the dark. Samples were then mounted and imaged using a Leica SP8 confocal microscope with a 40× objective.

Fluorescence intensity was quantified using ImageJ software, with corrected total cell fluorescence (CTCF) calculated as: Integrated Density/(Cell Area × Mean Background Fluorescence).

Etoposide, a positive control (a final concentration of 50 µM, Etop), was used to induce DNA DSBs.

### Immunofluorescence of formalin-fixed paraffin-embedded human trachea

Irradiated-human trachea samples and controls were fixed in 4% neutral buffered formalin overnight at RT, processed using a standard automated tissue processor, and embedded in paraffin wax. Sections (5–8 μm) were cut for labelling experiments.

Slides were dewaxed sequentially in xylene, ethanol gradients (100%, 90%, 70%, 40%), and dH_2_O, then transferred to citrate buffer (pH 6.0) for antigen retrieval via microwave heating (20 min). After cooling (30 min) and rinsing in running water (15 min), slides were placed in PBS to prevent drying. Tissue sections were framed with a PAP pen and blocked overnight at 4 °C in PBS containing 1% BSA, 10% FBS, 0.1% Triton X-100, and 0.3% Saponin.

After washing with PBS, slides were incubated with primary antibody: anti-γH2AX(Ser139) or anti-(6 -4) DNA photoproducts in immunofluorescence buffer (PBS with 3% BSA, 1% goat serum, 0.1% Triton X-100, 0.1% Saponin) overnight at 4 °C. Each primary antibody was incubated with a different tissue cut. A small parafilm cover was used to prevent drying. After washing with 0.5% Tween 20 in PBS, slides were incubated with secondary antibody (Alexa Fluor^TM^488 anti-Rabbit IgG or Alexa Fluor^TM^594 anti-Rabbit IgG, 1:1000, respectively) and Hoechst 33,342 (1:1000) for 1 h at RT in the dark.

Following washes with 0.5% Tween 20 in PBS, Vector TrueVIEW reagent was applied (5 min) to reduce autofluorescence. Slides were mounted with Vectashield Vibrance Antifade Mounting Medium and imaged using a Leica SP8 confocal microscope with a 40x objective. Image reconstruction was performed with ImageJ software.

### Annexin V apoptosis detection

Irradiated cells and controls were incubated in a humidified atmosphere at 37 °C with 5% CO_2_ for 12 h. The medium was aspirated and preserved for the identification of apoptotic cells. The adherent cells were washed with 2 mL of PBS. After washing, cells were incubated with 0.5 mL of TrypLE Express/without Phenol Red for 1 min at RT. After adding 2 mL of PBS, a cell suspension, including non-adherent and adherent cells, was centrifugated at 400 g for 5 min. For each treatment 100 µL of cell suspension (1 × 10^6^ cells) was mixed with FITC Annexin V (2.5 µL) and Propidium Iodide Solution (1 µL, PI), a viability dye, and incubated for 15 min at RT protected from light. The cells were washed with 400 µL of Annexin V Binding Buffer and centrifugated at 400 g for 5 min. The cells were resuspended in 250 µL of Annexin V Binding Buffer and analyzed by Cytek Aurora (Cytek Biosciences). The flow cytometry data was analyzed by FlowJo software. Staurosporine, a positive control (a final concentration of 1.25 µM, ST), was used to induce a non-lytic apoptosis.

### Crystal violet assay

To assess the effect of Far-UVC exposure on cell viability, cells were seeded into six-well plates at 0.35 × 10^6^ cells per well the day before irradiation, reaching 70–80% confluence at the time of irradiation. Cells were incubated in 500 µL of phenol red-free DMEM before being exposed to a specific Far-UVC dose and wavelength. After irradiation, the medium was replaced with fresh DMEM (2 mL per well), and cells were incubated at 37 °C with 5% CO_2_ for the required duration.

Cells were then washed twice with PBS, fixed in 4% paraformaldehyde for 15 min at RT, and stained with 1 mL of 0.5% crystal violet (CV) in 20% methanol for 20 min at RT. After staining, cells were washed four times with dH_2_O, air-dried, and solubilized in 1 mL of 30% acetic acid solution. The plate was incubated for 20 min with occasional shaking. Optical density (OD) was measured at 570 nm using a Synergy H1 Hybrid Reader (BioTek Instruments, Winooski, VT, USA). Etoposide (a final concentration of 50 µM, Etop) was used as a positive control.

### Lactate dehydrogenase (LDH) cytotoxicity assay

Cellular LDH released into the cellular medium upon plasma membrane damage by 222 nm light was assessed with CyQuant LDH Cytotoxicity assay kit according to the manufacturer’s instructions. The absorbance of LDH (OD) was measured at 490 nm using a Synergy H1 Hybrid Reader (BioTek Instruments, Winooski, VT, USA). Etoposide (a final concentration of 50 µM, Etop) was used as a positive control.

### Statistical analysis

Statistical analysis was carried out using GraphPad Prism10. Statistical differences were determined via one-way ANOVA with comparison based on a control column. Independent experiments were performed at least three times or otherwise as stated. Results are expressed as the mean ± SD. p-value < 0.05 was considered significant. *p < 0.05, **p < 0.01, ***p < 0.001, ****p < 0.0001, respectively.

## Electronic supplementary material

Below is the link to the electronic supplementary material.

Supplementary Material 1

## Data Availability

The data presented in this study are available on request from the corresponding author.

## Declarations

### Experimental model and study participant details

The studies involving human tissues and informed consent were reviewed and approved by London—Central Research Ethics Committee (REC) (REC No: 16/LO/1883) and by NHS Lothian SAHSC Bioresource (REC No: 20/ES/0061).

## References

[1] Kitagawa, H. et al. Effectiveness of 222-nm ultraviolet light on disinfecting SARS-CoV-2 surface contamination. *Am. J. Infect. Control*. **49**, 299–301. 10.1016/j.ajic.2020.08.022 (2021). 32896604 10.1016/j.ajic.2020.08.022 PMC7473342

[2] Buonanno, M., Welch, D., Shuryak, I. & Brenner, D. J. Far-UVC light (222 nm) efficiently and safely inactivates airborne human coronaviruses. *Sci. Rep.* **10**, 10285. 10.1038/s41598-020-67211-2 (2020). 32581288 10.1038/s41598-020-67211-2 PMC7314750

[3] Song, B. M. et al. Ultraviolet-C light at 222 Nm has a high disinfecting spectrum in environments contaminated by infectious pathogens, including SARS-CoV-2. *PLoS One*. **18**, e0294427. 10.1371/journal.pone.0294427 (2023). 38015931 10.1371/journal.pone.0294427 PMC10684113

[4] Sesti-Costa, R. et al. UV 254 Nm is more efficient than UV 222 Nm in inactivating SARS-CoV-2 present in human saliva. *Photodiagnosis Photodyn Ther.* **39**, 103015. 10.1016/j.pdpdt.2022.103015 (2022). 35843562 10.1016/j.pdpdt.2022.103015 PMC9281457

[5] Narita, K. et al. 222-nm UVC inactivates a wide spectrum of microbial pathogens. *J. Hosp. Infect.*10.1016/j.jhin.2020.03.030 (2020). 10.1016/j.jhin.2020.03.030 32243946

[6] Kang, J. W., Kim, S. S. & Kang, D. H. Inactivation dynamics of 222 Nm krypton-chlorine excilamp irradiation on Gram-positive and Gram-negative foodborne pathogenic bacteria. *Food Res. Int.* **109**, 325–333. 10.1016/j.foodres.2018.04.018 (2018). 29803456 10.1016/j.foodres.2018.04.018

[7] Taylor, W. et al. DNA damage kills bacterial spores and cells exposed to 222-Nanometer UV radiation. *Appl. Environ. Microbiol.* **86**10.1128/AEM.03039-19 (2020). 10.1128/AEM.03039-19 PMC7117916 32033948

[8] Naito, K., Sawadaishi, K. & Kawasaki, M. Photobiochemical mechanisms of biomolecules relevant to germicidal ultraviolet irradiation at 222 and 254 Nm. *Sci. Rep.* **12**, 18217. 10.1038/s41598-022-22969-5 (2022). 36309578 10.1038/s41598-022-22969-5 PMC9617911

[9] Pfeifer, G. P., You, Y. H. & Besaratinia, A. Mutations induced by ultraviolet light. *Mutat. Res.* **571**, 19–31. 10.1016/j.mrfmmm.2004.06.057 (2005). 15748635 10.1016/j.mrfmmm.2004.06.057

[10] Rastogi, R. P., Richa, Kumar, A., Tyagi, M. B. & Sinha, R. P. Molecular mechanisms of ultraviolet radiation-induced DNA damage and repair. *J. Nucleic Acids*. **2010**, 592980. 10.4061/2010/592980 (2010). 10.4061/2010/592980 PMC3010660 21209706

[11] Hessling, M., Haag, R., Sieber, N. & Vatter, P. The impact of far-UVC radiation (200–230 nm) on pathogens, cells, skin, and eyes—a collection and analysis of a hundred years of data. *GMS Hyg. Infect. Control*. **16**, Doc07. 10.3205/dgkh000378 (2021). 10.3205/dgkh000378 PMC7894148 33643774

[12] Buonanno, M. et al. Germicidal efficacy and mammalian skin safety of 222-nm UV light. *Radiat. Res.* **187**, 483–491. 10.1667/RR0010CC.1 (2017). 28225654 10.1667/RR0010CC.1 PMC5552051

[13] Yamano, N. et al. Evaluation of acute reactions on mouse skin irradiated with 222 and 235 Nm UV-C. *Photochem. Photobiol*. **97**, 770–777. 10.1111/php.13384 (2021). 33480023 10.1111/php.13384 PMC8359381

[14] Fukui, T. et al. Safety of 222 Nm UVC irradiation to the surgical site in a rabbit model. *Photochem. Photobiol*. **98**, 1365–1371. 10.1111/php.13620 (2022). 35313036 10.1111/php.13620 PMC9790646

[15] Narita, K. et al. Disinfection and healing effects of 222-nm UVC light on methicillin-resistant Staphylococcus aureus infection in mouse wounds. *J. Photochem. Photobiol. B*. **178**, 10–18. 10.1016/j.jphotobiol.2017.10.030 (2018). 29101868 10.1016/j.jphotobiol.2017.10.030 PMC5771808

[16] Kaidzu, S. et al. Evaluation of acute corneal damage induced by 222-nm and 254-nm ultraviolet light in Sprague-Dawley rats. *Free Radic. Res.* **53**, 611–617. 10.1080/10715762.2019.1603378 (2019). 30947566 10.1080/10715762.2019.1603378

[17] Ong, Q., Wee, W., Dela Cruz, J., Teo, J. W. R. & Han, W. 222-Nanometer Far-UVC exposure results in DNA damage and transcriptional changes to mammalian cells. *Int. J. Mol. Sci.* **23**10.3390/ijms23169112 (2022). 10.3390/ijms23169112 PMC9408858 36012379

[18] Nishikawa, J. et al. Far-Ultraviolet light at 222 Nm affects membrane integrity in monolayered DLD1 Colon cancer cells. *Int. J. Mol. Sci.* **25**10.3390/ijms25137051 (2024). 10.3390/ijms25137051 PMC11241724 39000160

[19] Al-Sadek, T. & Yusuf, N. Ultraviolet radiation biological and medical implications. *Curr. Issues Mol. Biol.* **46**, 1924–1942. 10.3390/cimb46030126 (2024). 38534742 10.3390/cimb46030126 PMC10968857

[20] Morkunas, V. et al. DNA-Damaging effect of different wavelength (206 and 257 nm) femtosecond laser pulses. *Photobiomodul. Photomed. Laser Surg.* **37**, 254–261. 10.1089/photob.2018.4540 (2019). 31050961 10.1089/photob.2018.4540

[21] Chandrasekhar, D. & Van Houten, B. In vivo formation and repair of cyclobutane pyrimidine dimers and 6 – 4 photoproducts measured at the gene and nucleotide level in *Escherichia coli*. *Mutat. Res.* **450**, 19–40. 10.1016/s0027-5107(00)00014-2 (2000). 10838132 10.1016/s0027-5107(00)00014-2

[22] Tavares, R. S. N. et al. Different biological effects of exposure to far-UVC (222 nm) and near-UVC (254 nm) irradiation. *J. Photochem. Photobiol. B*. **243**, 112713. 10.1016/j.jphotobiol.2023.112713 (2023). 37086566 10.1016/j.jphotobiol.2023.112713

[23] Schwarz, T. UV light affects cell membrane and cytoplasmic targets. *J. Photochem. Photobiol. B*. **44**, 91–96. 10.1016/S1011-1344(98)00126-2 (1998). 9757589 10.1016/S1011-1344(98)00126-2

[24] Panzures, A. 222-nm UVC light as a skin-safe solution to antimicrobial resistance in acute hospital settings with a particular focus on methicillin-resistant Staphylococcus aureus and surgical site infections: a review. *J. Appl. Microbiol.* **134**10.1093/jambio/lxad046 (2023). 10.1093/jambio/lxad046 36869801

[25] Narita, K., Asano, K., Morimoto, Y., Igarashi, T. & Nakane, A. Chronic irradiation with 222-nm UVC light induces neither DNA damage nor epidermal lesions in mouse skin, even at high doses. *PLoS One*. **13**, e0201259. 10.1371/journal.pone.0201259 (2018). 30044862 10.1371/journal.pone.0201259 PMC6059456

[26] Kaidzu, S. et al. Safety evaluation of Far-UV-C irradiation to epithelial basal cells in the corneal limbus. *Photochem. Photobiol*. **99**, 1142–1148. 10.1111/php.13750 (2023). 36437576 10.1111/php.13750

[27] Hung, K. F., Sidorova, J. M., Nghiem, P. & Kawasumi, M. The 6 -4 photoproduct is the trigger of UV-induced replication blockage and ATR activation. *Proc. Natl. Acad. Sci. U. S. A*. **117**, 12806–12816. 10.1073/pnas.1917196117 (2020). 32444488 10.1073/pnas.1917196117 PMC7293618

[28] Brahms, C. & Travers, J. C. Efficient and compact source of tuneable ultrafast deep ultraviolet laser pulses at 50 khz repetition rate. *Opt. Lett.* **48**, 151–154. 10.1364/OL.480103 (2023). 36563391 10.1364/OL.480103

