# Supplementary material for: Far-ultraviolet light causes direct DNA damage in human lung cells and tissues
Source: Sci Rep. 2025 May 24;15:18055. doi: 10.1038/s41598-025-02869-0 (PMC12102315; doi:10.1038/s41598-025-02869-0)
Supplement: Supplementary file 1 — Supplementary Material 1 [file 41598_2025_2869_MOESM1_ESM.doc]

**Supplementary Figures**


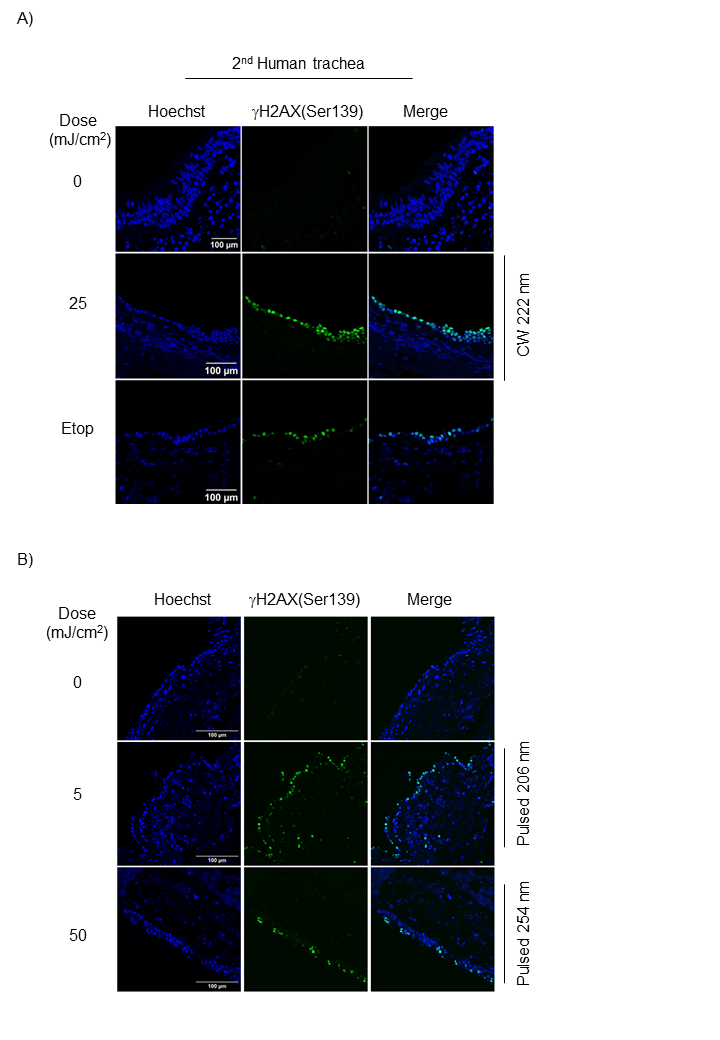


**Figure S1. CW 222 nm and pulsed 206 nm irradiation induced the phosphorylation of H2AX(Ser139) in human trachea.** (A) Representative immunofluorescence images of γH2AX(Ser139) staining (green) in a second set of human trachea after CW 222 nm irradiation Hoechst nuclei (blue). Scale bar, 100 μm. (B) Representative immunofluorescence images of γH2AX(Ser139) staining (green) after pulsed 206 nm irradiation in human trachea. Hoechst nuclei (blue). Pulsed 254 nm (50 mJ/cm^2^) irradiation was used as a positive control. Scale bar, 100 μm.


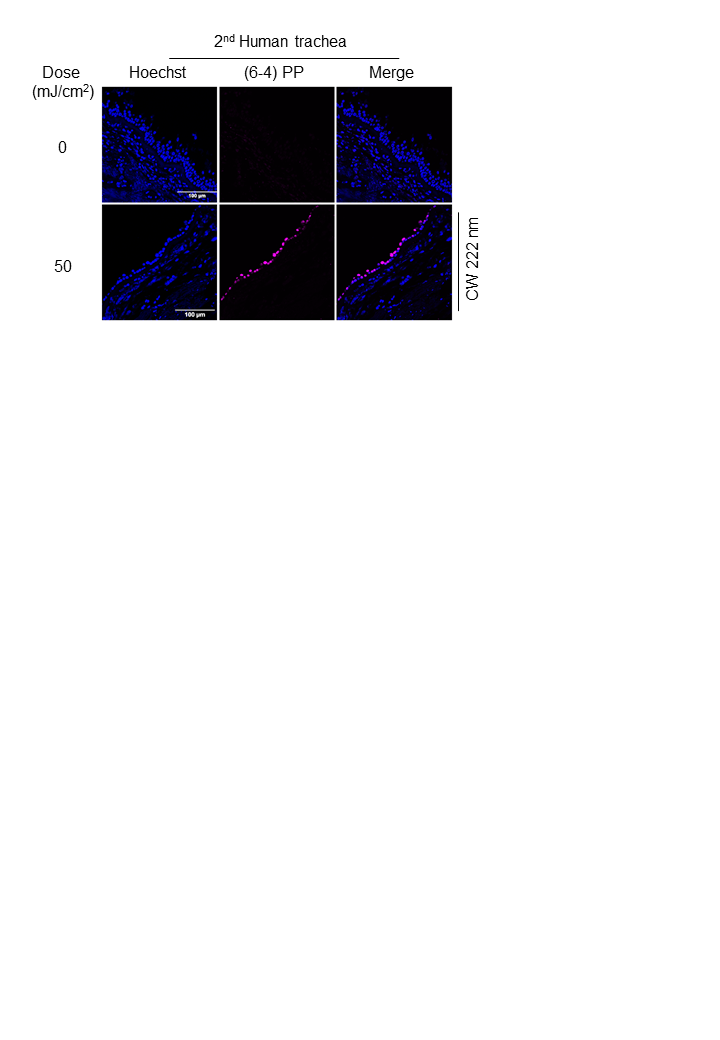


**Figure S2. (6-4) PP DNA lesions were detected in CW 222 nm-irradiated human trachea.** (6-4) PP staining (magenta) in a second set of human trachea after CW 222 nm irradiation. Hoechst nuclei (blue). Scale bar, 100 μm.

**Table S1**. **Key resources table**

| **REAGENT OR RESOURCE** | **SOURCE** | **IDENTIFIER** |
| --- | --- | --- |
| Antibodies | | |
| γH2AX(Ser139) | Cell Signaling Technology | 9718 |
| (6-4) DNA photoproducts [64M2]  We used as (6-4) PP abbreviation. | 2BScientific | 64M2 |
| Goat Alexa Fluor^TM^488 anti-Rabbit Ig | Thermo Fisher Scientific, Invitrogen | A-11034 |
| Goat Alexa Fluor^TM^594 anti-Rabbit Ig | Thermo Fisher Scientific, Invitrogen | A-21235 |
| Biological samples |  |  |
| Human tissue/trachea | London – Central Research Ethics Committee (REC) and facilitated by NHS Lothian SAHSC Bioresource | REC No: 16/LO/1883 and REC No: 20/ES/0061 |
| Chemicals, peptides, and recombinant proteins | | |
| Crystal violet | Med Chem Express | HY-B0324A/CS-2365 |
| Fetal bovine serum | Thermo Fisher Scientific | 10500064 |
| Penicillin/Streptomycin | Thermo Fisher Scientific | 15140122 |
| L-Glutamine | Gibco | 25030-024 |
| Staurosporine | Sigma | S4400 |
| Critical commercial assays | | |
| CyQuant&trade LDH Cytotoxicity assay kit | Invitrogen, Life Technologies | C20300 |
| FITC Annexin V Apoptosis Detection Kit with PI | BioLegend | 640914 |
| Experimental models: Cell lines | | |
| Beas-2B | American Type Culture Collection (ATCC), Manassas, VA, USA | CRL-3588 |
| NCI-H1299 | ATCC, VA, USA | CRL-5803 |
| HaCaT | ATCC, VA, USA | PCS-200-011 |
| Software | | |
| ImageJ | National Institute of Health, Bethesda, Maryland, USA | https://imagej.nih.gov/ij |
| ANOVA | SAS Institute Inc., Cary, NC, USA |  |
| GraphPad Prism v10 | Inc., La Jolla, Ca, USA |  |
| Flow Jo v10.8.1 | BD life Science |  |
| Other | | |
| DMEM/medium | Thermo Fisher Scientific | 21969-035 |
| DMEM (a phenol red-free medium) | Life Technologies | 31053044 |
| Hank’s Balanced Salt Solution | Capricorn | HBSS-2A |
| Hoechst33342/stain | Invitrogen, Life Technologies | 62249 |
| Vector TrueVIEW reagent | Vector Laboratories | SP8400 |
| Vectashield Vibrance Antifade Mounting medium | 2BScientific | H1700-10 |
| 222 nm system with integrated an Ushio B1 lamp module | johnellison.co.uk | Model: J8080, serial number: J8080-0001 |
| Optical power meter | Thorlabs | P1002909 |
